# Rapid haplotype inference for nuclear families

**DOI:** 10.1186/gb-2010-11-10-r108

**Published:** 2010-10-29

**Authors:** Amy L Williams, David E Housman, Martin C Rinard, David K Gifford

**Affiliations:** 1 Computer Science and Artificial Intelligence Laboratory, Massachusetts Institute of Technology, 32 Vassar Street, Cambridge, MA, 02139, USA; 2 David H. Koch Institute for Integrative Cancer Research at MIT, Massachusetts Institute of Technology, 40 Ames Street, Cambridge, MA, 02142, USA

## Abstract

Hapi is a new dynamic programming algorithm that ignores uninformative states and state transitions in order to efficiently compute minimum-recombinant and maximum likelihood haplotypes. When applied to a dataset containing 103 families, Hapi performs 3.8 and 320 times faster than state-of-the-art algorithms. Because Hapi infers both minimum-recombinant and maximum likelihood haplotypes and applies to related individuals, the haplotypes it infers are highly accurate over extended genomic distances.

## Background

The emergence of high throughput genotyping technologies has enabled rapid, low-cost assays of single nucleotide polymorphisms (SNPs) in large datasets of human subjects. These genotype data provide two unordered allele values at each queried genomic position, with each allele derived from the two homologous chromosomes in a diploid cell. However, genotype data do not identify which variant is present on each homologous chromosome.

A haplotype is an assignment of each allele to the homologous chromosome it resides on, and the haplotypes of a set of individuals can be determined, with varying levels of accuracy, from their genotype data using haplotype inference or 'phasing' techniques. Haplotypes are essential for many important genetic applications, including: (1) imputation of genotypes at loci that were originally untyped in a set of samples [1-5], a technique that can uncover novel disease susceptibility loci when incorporated into a genome-wide association study; (2) studying the results of meiosis - within a single generation or averaged across many generations - providing the opportunity to build genetic maps [6], identify recombination hotspots [7], and identify genetic causes of recombination rate variation [8]; (3) studying parental transmission effects such as imprinting [9]; (4) identifying signatures of selection [10], and many others. Indeed, much research at the frontier of biological understanding, such as the allelic control of chromatin structure, will require accurate haplotype information.

Genome scale haplotypes cannot be discovered using direct molecular means at present, so computational methods must be used to infer them. Algorithms for inferring haplotypes can be separated into three classes. One class of haplotyping algorithms applies to unrelated individuals, and techniques of this class use probabilistic constraints governed by mathematical models of population dynamics to infer haplotypes. Available algorithms [11,12] include PHASE [13], BEAGLE [3,4], HAPLOTYPER [14], and HAP2 [15,16]. The models these algorithms approximate are often insufficient to prevent switch errors - that is, positions with incorrectly assigned haplotypes relative to the previous heterozygous locus [13,16] - except across short genomic distances, as was recently demonstrated experimentally [17]. Additionally, haplotypes inferred from unrelated individuals can only reveal information about the results of meiosis (including the location of hotspots) averaged across thousands of generations and both genders.

The second class of haplotyping algorithms applies to individuals with known family relationships [18-26]. These algorithms infer haplotypes using the laws of Mendelian inheritance and the fact that allelic variants in close proximity to each other segregate together (that is, exhibit genetic linkage). Haplotypes inferred from family-based data are accurate across extended genomic distances: depending on the family size, they will contain few or no switch errors. Additionally, these datasets and algorithms enable the identification of the probable sites of *de novo *meiotic recombinations and gene conversions (which appear as short double crossovers), and have been used to build genetic maps of recombination rates [6], and identify hotspots [7]. Considering *de novo *meiotic recombinations and gene conversions enables the study of differences in location and number [27] of such events between individuals, including gender-based differences, and a gene affecting individuals' genome wide recombination rates has been identified [8]. Importantly, haplotypes from family-based datasets are also used to perform linkage analysis to study the genetic basis of disease within families.

The third class of haplotyping algorithms applies to many family trios which contain data for a father, mother, and one child; approaches in this class leverage techniques from the other two classes outlined above. In particular, algorithms for haplotyping trio data use the laws of Mendelian inheritance to resolve the haplotypes of the trio individuals at every locus where one of the individuals is homozygous. For the remaining ambiguous loci, these algorithms utilize the mathematical models that govern haplotype expectations for unrelated individuals, with adaptations to apply to trio data. PHASE [13], BEAGLE [3,4], HAP2 [15,16], and other algorithms support this form of haplotyping. Trio-based approaches produce haplotypes with far fewer switch errors than techniques that rely only on data from unrelated individuals. However, haplotypes from trios still do not provide information about *de novo *meiotic recombinations or gene conversions, and therefore suffer from the same limitations for studies of the results of meiosis as do haplotypes from unrelated individuals.

Hapi is a new dynamic programming algorithm that infers both minimum-recombinant and maximum likelihood haplotypes, and performs substantially faster than all other haplotyping algorithms for the nuclear family problem. Nuclear family derived genotypic data identifies parents and their children, but provides no information about relationships within a larger pedigree. Minimum-recombinant haplotypes assign family members' genotypes to homologs such that the number of recombinations that occur in the homologs the parents transmitted to the children is minimized. Maximum likelihood haplotypes utilize recombination frequencies between successive loci from a genetic map to calculate the most likely haplotype reconstruction.

Maximum likelihood haplotypes are often substantially similar or identical to minimum-recombinant haplotypes. Both approaches to haplotype estimation have strengths and weaknesses.

Minimum-recombinant haplotyping may yield suboptimal results when the recombination frequencies between loci in some region varies widely. (Recombination rate variation can occur if the distance between pairs of loci varies dramatically within a region, or, if genotypes are sampled at a very fine scale, recombination hotspots and coldspots can produce such variation.) Maximum-likelihood haplotyping reports only the most likely haplotype, a feature that can be misleading to a user when the difference in probability to alternate haplotypes is small. Typically this occurs when the number of recombinations across the alternate haplotypes are the same, and in such a case, minimum-recombinant haplotyping reports the ambiguities. Historically, geneticists have manually performed minimum-recombinant haplotype assignment to analyze small datasets. Hapi enables this approach to be applied to the very large datasets currently produced by high-throughput SNP genotyping.

Several existing programs for haplotyping related individuals are based on the Lander-Green algorithm [19], including Merlin [20], GENEHUNTER [21,22], and Allegro [23,24]. The basic approach of the Lander-Green algorithm uses hidden Markov models (HMMs) to obtain a probability distribution of haplotype assignments for individuals in a pedigree. A user can either sample a haplotype from this distribution, or, more commonly, obtain the maximum likelihood haplotype assignment. The state space for these HMMs is composed of inheritance vectors at each locus that are bit strings encoding which chromosome homolog a parent transmitted for each child in the pedigree at that locus. This state space is inherently exponential, with 2^2*n *^possible values, where *n *is the number of non-founders or individuals with at least one parent in the pedigree.

Although Merlin, GENEHUNTER, and Allegro all employ techniques to reduce computational space and time requirements of this basic algorithm, all are relatively inefficient; in general, each requires exponential time in the number of non-founders in the pedigree. One technique that all these algorithms employ is to avoid representing inheritance vectors that are inconsistent with Mendelian inheritance. In addition, Merlin [20] uses sparse gene flow trees that avoid redundant representations for states with identical likelihoods or a probability of zero. Allegro [24] uses multi-terminal binary decision diagrams (MTBDDs) [28], which are more general than sparse gene flow trees. MTBDDs are at least as sparse as Merlin's sparse gene flow trees, and depending on how they are constructed, can be smaller. The optimized representations that Merlin and Allegro utilize are effective in reducing the number of states at a single locus. However, transition probabilities will, in general, differ for most or all possible transitions between states at adjacent loci. Because of this, the algorithms must represent most or all of the 2^2*n *^states in order to perform multipoint analyses, including haplotyping.

Superlink [29] is another maximum likelihood haplotyping algorithm that uses Bayesian networks. While Superlink employs several optimizations to improve its efficiency, it performed slower than Merlin and Allegro in our experiments.

Hapi's optimizations reduce the state space that it must examine both at a single locus and across multiple loci, as it is able to avoid considering transitions between all possible states at adjacent loci. The optimizations we introduce in Hapi represent a leap forward in reducing the algorithm runtime and space complexity compared to existing algorithms. The following is a summary of Hapi's optimizations; further details appear in Materials and methods:

1. When a parent *p *is homozygous at a locus *l*, Hapi only builds states for *l *in which the homolog that parent *p *transmitted does not exhibit recombination relative to the previous locus. In connection with this, Hapi does not build states at loci where both parents are homozygous since recombination cannot be observed at these loci. This optimization is natural for minimum-recombinant haplotyping, but it requires special consideration in the context of maximum likelihood haplotypes.

2. At loci where Mendelian inheritance cannot unambiguously infer for a set of children which parent transmitted each allele, Hapi uses a novel, concise representation of the ambiguities instead of forming an exponential number of states for all possible transmissions to the children. Hapi also avoids building any states that represent recombinations on both homologs for the ambiguous children and later evaluates whether that decision is consistent with nearby loci.

3. To transition between states at adjacent loci, Hapi considers a state at the previous locus as possibly transitioning to either two or four states at the next locus, depending on the genotypes and possible phase assignments of the parents at that locus. This optimization is actually a by-product of the first two optimizations mentioned above, but deserves separate consideration. Normally if two adjacent loci each have *s *states, there are *s*^2 ^possible state transitions (note that *s *may be an exponential number). Kruglyak and Lander [30] introduced a fast Fourier transform optimization that reduced the computational burden for transiton calculations to *O*(*s*·log *s*), but Hapi's transition runtime is only *O*(*s*), that is, linear in the number of states at a locus.

4. Some states encode the same transmissions of homologs from the parents to the children and differ only in the parents' phase. These states are equivalent downstream of the current locus and Hapi only retains the state with minimum recombinations or maximum likelihood. Kruglyak *et al*. [22] first discovered a more general form of this optimization that applies to all founders in a pedigree. Hapi applies this optimization to parents in a nuclear family.

5. The previous optimization is most effective when none of the children are missing genotype data. We devised a mechanism for comparing nearly equivalent states in the presence of children with missing data that often enables the detection and elimination of suboptimal states.

6. At each locus, Hapi only considers states that are consistent with Mendel's laws for the genotypes of the individuals and spends no time processing any inconsistent states. Other algorithms also employ similar optimizations that help reduce the number of states they examine [20,21,24].

To demonstrate the efficacy of Hapi's optimizations in the context of real genotype data, we ran Merlin, Allegro, Superlink, PedPhase 2.0 [26] and Hapi on a dataset containing 103 nuclear families. In these experiments, Hapi ran 3.8 and 320 times faster than Merlin, and provided even greater runtime improvements over Allegro, Superlink, and PedPhase (see Results).

Existing algorithms have limits on the size and number of families they can haplotype. Hapi makes possible the efficient haplotyping of very large numbers of families as well as families with large numbers of individuals. Because of the relative ease of gathering genotypes for nuclear families, we expect that the number of nuclear families within datasets will continue to grow and that Hapi will provide the opportunity to haplotype this large quantity of data. The techniques Hapi implements to efficiently haplotype nuclear families also apply to general pedigrees, and thus promise to extend the size of pedigree datasets beyond the limitations of roughly 20 non-founders inherent in existing algorithms (see Conclusions). Hapi is freely available for non-profit use [31].

The remainder of this paper describes our experimental results (Results and discussion), gives a summary of our contributions (Conclusions), and describes our algorithm in detail (Materials and methods).

## Results and discussion

We have evaluated Hapi's runtime performance compared to three state-of-the-art algorithms: Merlin [20], Allegro [24], and Superlink [29], programs in current use for family-based haplotype assignment. Like most algorithms for computing maximum likelihood haplotypes, these programs have exponential complexity in general. However, each contains several optimizations, and these are the most suitable programs for comparison to Hapi. We omitted GENEHUNTER from our comparison because Merlin outperforms it [20]. We ran each program on a dataset of nuclear families derived from a pedigree from the Huntington's Disease Venezuela Collaborative Study [32]. This Venezuelan pedigree has 757 individuals and 458 families. None of Merlin, Allegro, or Superlink can successfully haplotype such a large pedigree. Hapi can currently only analyze nuclear families where both parents have genotype data, so the pedigree was broken up into such families. The choice to break up such a large pedigree into smaller sets of related individuals is necessary regardless of which haplotyping tool is used since runtime and memory requirements impose hard limits on the scalability of existing algorithms.

The derived nuclear family dataset contains 103 nuclear families where both parents have data. These families have a total of 438 individuals. Note that because we analyzed the families separately, we double counted individuals that appear in more than one family (for example, as a parent in one and a child in another, or as a parent in more than one family).

These families range in size from one to eleven children, with an average of 2.23 children per family. There are 86 families with three or fewer children (308 total individuals), with an average of 1.56 children for that subset of families. Using the Illumina linkage IV_v3 SNP panel, genotypes at 5,456 SNPs covering the whole genome were obtained for each individual in the dataset [32]. The numbers of SNPs per chromosome are roughly proportional to the chromosome's size and range from 102 on chromosome 21 to 468 on chromosome 2. Prior to analysis, the PEDSTATS [33] and PedCheck [34] programs were used to remove genotypes exhibiting non-Mendelian errors. When processing a family, Hapi omits loci that are missing data for either parent, but the missing data status of one family does not affect any other family in the dataset.

Table 1 shows timing results from our experiments of performing maximum likelihood haplotyping using Hapi, Merlin, Allegro v2, and Superlink on a 2.30 GHz AMD Opteron machine with 32 GB of RAM. Although this is a multi-core processor, none of the algorithms are parallelized, so their runtimes are directly comparable. We used Hapi to infer maximum likelihood rather than minimum-recombinant haplotypes in this set of experiments because the other programs address that problem, and because that form of haplotyping is slower in Hapi. All programs except for Superlink (see below) used less than 8 GB of memory.

**Table 1:** Runtime results comparing Hapi to other family-based haplotyping algorithms

		All families		≤3 Children	
			
Machine	Program	Runtime	Speedup	Runtime	Speedup
	Hapi	3.112 s	-	2.225 s	-
2.30 GHz	Merlin	1005 s	323×	8.662 s	3.84×
AMD Opteron	Allegro v2	7661 s	2,462×	14.50 s	6.43×
	Superlink	1393 s*	448×	38.75 s	17.2×

1.40 GHz	Hapi	4.732 s	-	3.451 s	-
Pentium M	PedPhase 2.0	>21,600 s (6 h)^†^	>4,500×	>21,600 s (6 h)^†^	>6,000×

Runtimes for maximum likelihood haplotyping using Hapi, Merlin Allegro and Superlink of nuclear families from the Huntington's Disease Venezuela Collaborative Study [32]. We list times for haplotyping all nuclear families and for haplotyping those with three or fewer children. *Superlink failed to haplotype the family with 11 children; we therefore used only 8 of the children from the 11 child family to time it. Times are averages from running Hapi eight times and Merlin, Allegro, and Superlink three times each. Runtimes also on a different machine for minimum-recombinant haplotyping using Hapi (averaged from eight runs) and PedPhase ^†^for chromosome 1 only.

Superlink ran for over six hours without finishing when we used it haplotype chromosome 1 for all families in the dataset. At that time, the program reported that 0% of the haplotyping was complete. We found that Superlink uses an excessive amount of memory (>24 GB) to haplotype a family with nine or ten children. The times for Superlink therefore reflect its haplotyping a modified set of families, with three of the children removed from the original eleven child family. Superlink used less than 8 GB of memory when analyzing this modified dataset.

We include times for haplotyping all families in the dataset (modified for Superlink), as well as the subset of families with three or fewer children in Table 1. Because of the fixed and disproportionate overhead involved in printing the haplotypes in Hapi and Merlin (approximately .5 seconds in Hapi or about 16% of runtime and approximately 29 seconds in Merlin or <3% of runtime), we report the times only for reading in the dataset and performing the haplotyping in these programs, but not printing the results. Source code is not publicly available for Superlink, so we could not modify it to avoid printing haplotypes, but such a change is unlikely to dramatically affect its runtime. We also did not modify Allegro to prevent it from printing haplotypes, but its runtime is also unlikely to change significantly compared to the current results. As Table 1 shows, Hapi is substantially faster than Merlin, running 323 times faster for the entire dataset and 3.84 times faster for the subset of families with three or fewer children. Hapi compares even more favorably against Allegro and Superlink, even though Superlink is only able to haplotype a reduced-sized dataset. When haplotyping the entire dataset, Hapi runs 2,462 times faster than Allegro and 448 times faster than Superlink's analysis of the smaller dataset. For haplotyping the subset of families with three or fewer children, Hapi runs 6.43 times faster than Allegro and 17.2 times faster than Superlink. Hapi's speedup for the entire dataset demonstrates experimentally the vast difference between the theoretical complexity of these algorithms. Whereas Merlin, Allegro, and Superlink have exponential runtime complexity, Hapi runs in polynomial time in practice (see Additional file 1 for complexity analysis). At the same time, the more modest gains for the families with three or fewer children is unsurprising. The other algorithms scale exponentially in the number of non-founders or, in the case of nuclear families, in the number of children in the family being analyzed. When that number is very small, an exponential algorithm will not differ as significantly from one that has polynomial runtime in practice. Our algorithm is still significantly faster than these programs even in this case that is less taxing to an exponential algorithm.

Besides these maximum likelihood systems, we compared Hapi's minimum-recombinant haplotyping to PedPhase 2.0, which uses an Integer Linear Programming algorithm to calculate minimum-recombinant haplotypes for pedigrees [26]. PedPhase 2.0 runs only in Windows, and we used a 1.40 GHz Pentium M laptop with 1.24 GB of RAM to compare runtimes of these two systems. Table 1 gives timing results on this machine for Hapi and PedPhase. We ran PedPhase on the entire dataset and on the families with three or fewer children. In both cases, PedPhase did not exceed available memory, and ran for over 6 hours without haplotyping even chromosome 1. Because 464 of the 5,456 total SNPs reside on chromosome 1, we estimate that the total runtime for PedPhase on this dataset would be at least 70 hours. In contrast, Hapi completes haplotyping the entire dataset in 4.732 seconds (in Linux) on this machine.

As we discuss in Additional file 1 the number of states in Hapi is affected by the number and pattern of markers that are missing data. Our nuclear family dataset contains only 1.17% missing data. To explore the runtime performance of Hapi in the presence of moderate to significant proportions of missing data, we modified it to randomly drop various proportions of data. Table 2 gives the results of our simulations. In the most extreme case of 50% missing data, Hapi's average runtime was 36.38 s, which is still 27.6 times faster than Merlin. Real datasets will generally contain 5% or less missing data, and we probabilistically dropped 3.83% markers from the original data to obtain approximately 5% missing data. In this scenario, Hapi performed only 5.21% slower compared to haplotyping the dataset without the added missing data (306 times faster than Merlin). These results demonstrate that Hapi is robust to haplotyping data with significant proportions of missing data and performs very well for the more modest missing data proportions for which it is likely to be used.

**Table 2:** Timing results from simulations of extreme amounts of missing data

Total % missing	Simulation probability	Runtime	Slowdown	Speedup vs. Merlin
5%	3.83%	3.274 s	5.21%	306×
10%	8.83%	3.564 s	14.5%	281×
20%	18.8%	4.567 s	46.8%	220×
30%	28.8%	6.897 s	122%	145×
40%	38.8%	11.36 s	265%	88.5×
50%	48.8%	36.38 s	1070%	27.6×

Hapi's runtime performance for haplotyping the dataset discussed in Results in the presence of various total proportions of missing data. Because this dataset contains 1.17% missing data already, we dropped genotypes according to the indicated probabilities in order to obtain the total overall proportions of missing data. The table lists the runtime, percentage slowdown compared to running Hapi on the unmodified dataset, and the speedup compared to running Merlin on the unmodified dataset.

**Figure 1:**
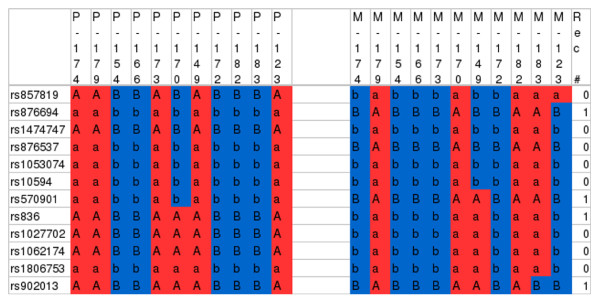
**Sample inheritance vector output from Hapi imported into a spreadsheet**. Output from Hapi showing the inherited homologs on chromosome 1 for a family with 11 children from the Huntington's Disease Venezuela Collaborative Study [32]. Hapi produces CSV format output, which we imported into a spreadsheet. To color the cells, we used conditional formatting based on the homolog value transmitted. The output of inheritance vector values uses letters A and B. Lower-case letters indicate the transmitting parent is homozygous and the presence of recombination unknown. Each column is labeled with the child's numerical id with either a 'P' or an 'M' preceding it to indicate either paternal or maternal-derived homologs. The left most column gives the SNP rs numbers, and the right most column lists the number of recombinations across all children at the given locus.

Hapi produces output in text or CSV format, suitable for import into a spreadsheet. It can output either the actual haplotypes with allele values or the children's inheritance vector values. The latter is useful for inspecting the results of meioses, including recombination patterns. Figure 1 shows the inheritance vector output from Hapi for a family with 11 children, imported into a spreadsheet. This output uses letter symbols rather than bit values, with lower case letters indicating that the corresponding meiosis is uninformative. To help identify recombinations sites, we use the spreadsheet program's conditional formatting feature to color the cells based on which homolog the child received. The output from Merlin, Allegro, and Superlink provide the same information as Hapi, but each of these programs uses its own text-based format. We expect that geneticists will find the ability to import Hapi's output into a spreadsheet to be more intuitive and more convenient than the output from other programs.

## Conclusions

Assignment of haplotypes is an important element in a number of significant areas of genetic analysis, including locating genes involved in human disease, analyzing the products of meiosis to locate recombination hotspots and gene conversions, and studying population dynamics and history for humans and other species. Because of their importance, researchers have developed computational algorithms for inferring haplotypes from genotypes. The most effective approach to this problem is to use data for individuals whose family relationships are known.

Inferring minimum-recombinant haplotypes for the individuals in a pedigree is known to be NP-hard in general [25,35]. Problems classified as NP-hard are not known to have a polynomial time (that is, efficient) solution, and are therefore thought to be computationally intractable. Existing algorithms computing either maximum likelihood (based on recombination rates) or minimum-recombinant solutions for pedigrees consequently have exponential complexity.

Hapi is an efficient algorithm for inferring both minimum-recombinant and maximum likelihood haplotypes for nuclear families. Hapi runs in polynomial time in practice (see Additional file 1 for algorithm complexity details), and our experimental data demonstrate the effectiveness of our approach. When haplotyping a large dataset of nuclear families, Hapi outperforms the state-of-the-art system Merlin with a speedup of between 3.8 and 320 times. Hapi also runs between 6.4 and 2,460 times faster than Allegro and between 17 and 448 times faster than Superlink.

The optimizations Hapi uses to efficiently haplotype nuclear families can also be extended to pedigrees. A detailed discussion of this problem is available elsewhere [36], but we give a brief description here. Two of Hapi's optimizations - eliminating equivalent states for all pedigree founders, and avoiding inheritance vectors that are inconsistent with Mendelian Inheritance - are already included in known algorithms. The other optimizations can apply individually to each of the nuclear families that make up the pedigree. Whenever one or both parents in one of the pedigree families is homozygous, it suffices to propagate the inheritance vector values corresponding to the parent(s) transmitted homologs from the states at the previous locus. (The system cannot skip uninformative loci for a particular family since other families in the pedigree will usually be informative.) Additionally, the ambiguous inheritance vectors optimization applies to all offspring in the pedigree except shared individuals that are a child in one family and a parent in another. In utilizing these optimizations, the system need only consider a linear number of transitions for the inheritance vectors corresponding to each nuclear family. Note that the algorithm must build states corresponding to all combinations of inheritance vector values across all the nuclear families. The bound on the number of states at each locus using our approach is therefore *O*((2^i* ^*s*)*^r^*), where *s *is the maximum states the algorithm would produce to evaluate any of the nuclear families individually, *r *is the number of nuclear families in the pedigree, and i* is the maximum number of shared individuals in any nuclear family. This bound, while exponential, compares favorably against the bound of *O*(2^2*n*-*f*^) states per locus of existing techniques since *r*·i* <*n *(note: there must be at least one offspring that is not a shared individual). With this reduction in the bound on the number of states, the optimizations Hapi employs make possible the haplotyping of larger pedigrees than can be handled by existing techniques.

As time passes and technology improves, genotype datasets will continue to grow in size, both numbers of individuals and numbers of loci assayed. As such, faster tools for haplotype analysis will be essential. Existing algorithms for haplotyping related individuals have hard limits on the size of families they can analyze because of their exponential complexity. These algorithms are consequently ineffective for datasets with thousands of families or for families with large numbers of children. Hapi provides a solution that is able to meet many of these future challenges.

## Materials and methods

Hapi performs both minimum-recombinant and maximum likelihood haplotyping for nuclear families. These two haplotyping approaches are similar, and we first present the minimum-recombinant algorithm. Later we will describe how to extend this approach to calculate maximum likelihood haplotypes. This paper describes an algorithm for haplotyping nuclear families that have genotype data for both parents and some number of children. We elsewhere describe how to generalize the algorithm to infer haplotypes for nuclear families with data for only one parent or to sets of siblings only (that is, without data for either parent) [36].

Hapi seeks to find a minimum-recombinant haplotype solution that is *globally *minimal across the chromosome length rather than *locally *minimal between successive pairs of loci. Thus, a solution may contain a locus that has an alternate assignment of individuals' alleles to homologous chromosomes that yields fewer recombinations from the previous locus (that is, locally), but not over the entire chromosome length (that is, globally). An example of such a locus from real data for a family of human subjects is described in the Example subsection.

Hapi uses inheritance vectors, represented using bit strings, to encode which chromosome homolog each parent transmitted to each child at a locus. These bit strings are composed of 2*c *bits, where *c *is the number of children in the nuclear family.

A dynamic programming equation for calculating minimum-recombinant haplotypes is given below. The function $R\left(l,\vec{v}\right)$ calculates the minimal number of recombinations necessary to reach inheritance vector $\vec{v}$ at locus *l*:

(1) $$R\left(l,\vec{v}\right)=\underset{\vec{w}}{\min}\left\{R\left(l-1,\vec{w}\right)+H\left(\vec{w},\vec{v}\right)\right\}.$$

Here, $R\left(l-1,\vec{w}\right)$ is the minimum number of recombinations necessary to reach an inheritance vector $\vec{w}$ at the previous locus *l*-1. $H\left(\vec{w},\vec{v}\right)$ is the number of recombinations between vectors $\vec{w}$ and $\vec{v}$, which is equal to the number of bits that differ between them, that is, the hamming distance. The initial number of recombinations at locus *l *= 0 is defined naturally as $R\left(l=0,\vec{v}\right)=0$.

A naive implementation of the above dynamic programming recurrence would initialize all 2^2*c *^possible inheritance vectors at locus *l *= 0 and would model most or all of these vectors at successive loci. Hapi functions differently: the initial locus has only one inheritance vector, and successive loci model a very small number of inheritance vectors.

Hapi uses a locus *state *to store the information computed in the above dynamic programming equation. A locus state stores: (1) an inheritance vector; (2) the assignment of the heterozygous parent's or parents' genotype alleles to homologs that is consistent with this inheritance vector; (3) the minimal number of recombinations necessary to reach this state/inheritance vector value; (4) a pointer to the state or states at the previous locus that yields this minimal number of recombinations; and (5) a bit string encoding which children have ambiguous inheritance values (necessary for some kinds of loci as we describe later). Because the parents' allele to homolog assignments imply part or all of the inheritance vector values, there is only one consistent parent assignment for each inheritance vector.

After evaluating equation (1) by building the necessary states for all loci, it is straight forward to deduce haplotypes. Hapi does this by performing the assignments of alleles to homologs as dictated by the minimum-recombinant state at the final locus and then back tracing to states at previous loci. Rather than waiting until the final locus to make these assignments and perform back tracing, Hapi does this work whenever a locus yields only one state (which happens frequently). The one state at that locus and those leading to it at previous loci are guaranteed to have minimum recombinations. Performing this process before the final locus allows the system to reclaim the memory used to store states.

**Figure 2:**
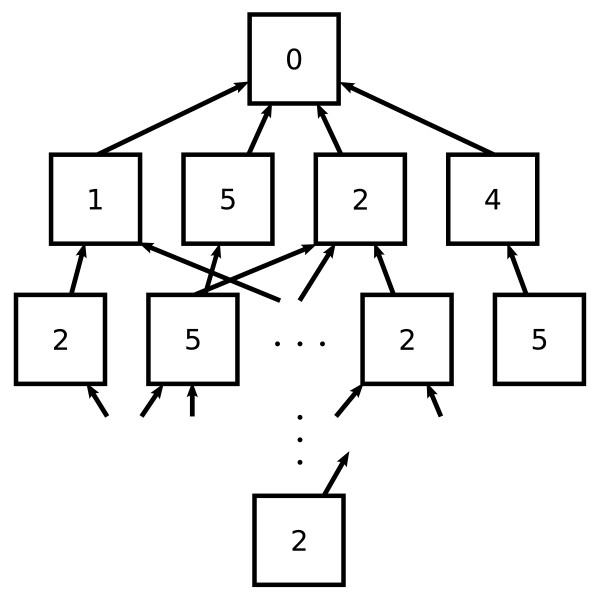
**Example graph of states across several loci**. A pictorial representation showing the relationship between states at different loci. Each row of boxes correspond to a locus; boxes represent a state and indicate the numbers of recombinations the state incurs; arrows point to previous state(s). Once the system deduces a single state at some locus - shown here as the bottom box - it back traces by traversing the pointers and assigns the haplotype values from the states it encounters. The numbers are not from real data.

We give an illustrative example of what a graph of states generated by our algorithm might look like in Figure 2. In this graph, boxes represent states, and each row of boxes corresponds to the states for a single locus. The number in each box represents the minimal number of recombinations necessary to reach that state. The first locus (top-most box) has only one box/state with an initial value of zero recombinations. At the second locus, there are four states that have between one and five recombinations. Note that at the third locus, the second state has pointers to two different states at the previous locus. The final locus has only one state. Once the system determines this final state, it performs back tracing along pointers to previous states, and uses the haplotype values stored in the encountered states to make the allele assignments.

Hapi implements six optimizations that allow it to very efficiently infer minimum-recombinant haplotypes, and it uses these same optimizations to calculate maximum likelihood haplotypes, as we describe later. The goal of each optimization is to reduce the number of states and state transitions that Hapi must consider and store. Below, we give details about five of the optimizations Hapi implements. The last optimization applies at loci where one or more children are missing data, a scenario we discuss later. Note that Hapi builds states for a locus based on the states at the previous locus and the genotypes of the individuals at the locus being considered. Considering states at the previous locus is necessary for two of Hapi's optimizations. The initial state for a chromosome cannot depend on previous locus states and is therefore built differently as we discuss later.

### Non-recombinant states for homozygous parents

When one or both of the parents at a locus are homozygous, which homolog the homozygous parent(s) transmitted is ambiguous. A naive implementation of the Lander-Green algorithm builds states corresponding to all possible homolog transmissions for the homozygous parent, yielding 2*^c ^*inheritance vector values for each homozygous parent. Instead of building and processing this exponential number of states, Hapi copies the inheritance vector values corresponding to the homozygous parent from the states at the previous locus. Typically the number of unique inheritance vector values for the homozygous parent at the previous locus is small, though it is possible for this number to be large. In general, other optimizations aid in keeping the number of states small, and our experimental results demonstrate that the number of states is small in practice.

This approach of copying inheritance vector values for the homozygous parent assumes a lack of recombination for this uninformative case, and this will always yield minimal recombinations. The next locus that is heterozygous for the parent in question will indicate if a recombination has occurred within any region of homozygosity for that parent.

For loci where both parents are homozygous, all 2^2*c *^possible inheritance vectors are consistent with the genotypes. Rather than building all states or copying every state from the previous locus, Hapi simply skips these loci. Subsequent loci utilize the states located at the most recent locus for which states exist. Table 3 gives an example from real data of a locus in which one parent is homozygous and the other parent is heterozygous. The inheritance vector values corresponding to the homozygous parent *p*_1 _are shown as the second element in each of the ordered pairs in the rows labeled $\vec{v}$. The inheritance vector values for the homozygous parent in the two states *a *and *b *are the same as those in the previous state since Hapi copies these values. Without this copying optimization, the locus would have 2·2*^c ^*states rather than two. Merlin [20] and Allegro [24] also include techniques that reduce the number of states they represent in the presence of uninformative meioses. These techniques represent redundancies in states' probabilities and are effective at a single locus, but transitions between states at adjacent loci inhibit their utility since differing transition probabilities typically reduce the amount of redundancy in the data.

**Table 3:** Two states at a fully informative for one parent locus built from the previous state

		Parents		Children					# Rec
				
		***p***_**0**_	***p***_**1**_	***c***_**0**_	***c***_**1**_	***c***_**2**_	***c***_**3**_	***c***_**4**_	
Prev	$\vec{v}$			〈0, 1〉	〈1, 1〉	〈1, 1〉	〈0, 0〉	〈1, 1〉	0
State	*hap*	〈a, g〉	〈a, a〉	〈g, a〉	〈a, a〉	〈a, a〉	a, a〉	〈a, a〉	
*a*	$\vec{v}$			〈**1**, 1〉	〈**0**, 1〉	〈**0**, 1〉 〈	0, 0〉	〈**0**, 1〉	4
State	*hap*	〈g, a〉	〈a, a〉	〈g, a〉	〈a, a〉	〈a, a〉 〈	a, a〉	〈a, a〉	
*b*	$\vec{v}$			〈0, 1〉	〈1, 1〉	〈1, 1〉 〈	**1**, 0〉	〈1, 1〉	1

An example locus with one heterozygous and one homozygous parent that shows one state at the previous locus and the two states Hapi builds based on this previous state. This example is from the real dataset discussed in Results. The rows labeled $\vec{v}$ show the states' inheritance vectors and the rows labeled *hap *give haplotype assignments of the alleles. Hapi copies the inheritance vector values corresponding to the homozygous parent from the previous state to states *a *and *b*. Recombinations result from differing inheritance vector values from the previous state; these differences appear in bold and the states' total number of recombinations appear in the right-most column. Note that the heterozygous parent's inheritance vector values in the two states are exactly opposite each other and are therefore equivalently labeled.

### Ambiguous inheritance vector values

At loci where both parents are heterozygous with the same genotype (which we later term 'partly informative'), a heterozygous child will have the same genotype as its parents. As a result, these heterozygous children are *a priori *ambiguous as to which parent transmitted each of their alleles: either parent could have transmitted either allele.

Existing algorithms build states corresponding to all possible inheritance vector values for these ambiguous children, and for a given assignment of the parents' alleles to homologs, each heterozygous child has two possible inheritance vector values. Thus, for *h *heterozygous children, there are 2*^h ^*possible inheritance vectors for each of the four possible assignments of parents' alleles to homologs, or 4·2*^h ^*total inheritance vectors/states consistent with the individuals' genotypes at these loci.

Instead of building this exponential number of states, Hapi again uses the states at the previous locus to reduce the number of states it must build. The system maps each previous state to four states corresponding to each assignment of parents' alleles to homologs. Note that multiple previous states can map to the same state, so the number of states usually does not quadruple. Also note that homozygous children have only one inheritance vector value that is consistent with a given assignment of parents' alleles, so they do not affect the number of necessary states.

Heterozygous children have two consistent inheritance vector values for a given assignment of parents' alleles to homologs, and these two values are opposite each other. If the inheritance value in the previous state is equivalent to one of these two values, Hapi uses the value equivalent to the previous state in the state being built. The other inheritance value results in two crossovers for the child, one from each parent. Such an event is extremely unlikely, yet if it were to take place, downstream loci that are fully informative would reveal its occurrence. In that rare case, Hapi will mark the partly informative locus as ambiguous during back tracing, since it is impossible to know whether these two recombinations took place at the earlier partly informative locus or at the later fully informative locus. (Maximum likelihood haplotyping determines the location of the recombinations based on recombination frequencies.)

In the case that the inheritance value in the previous state is not equal to one of the two ambiguous inheritance values, the previous inheritance value must differ from these two values in exactly one bit. For example, if the previous value is 〈0, 0〉 and is not equal to either of the values at the current locus, they must be 〈0, 1〉 and 〈1, 0〉. The differences between the two consistent values and the previous one represent a recombination in one or the other parent. Which parent recombined is ambiguous at this locus and can only be determined at later loci.

Rather than creating separate states for these two inheritance values - which would yield an exponential number of states across multiple children - Hapi instead marks the child as having ambiguous inheritance. A child's inheritance being marked as ambiguous means that its inheritance vector value can be inverted without inducing additional recombinations - both possibilities result in the exactly one recombination.

The choice of which of the two inheritance values to store in the state is arbitrary, and Hapi indicates that a child is ambiguous using another bit vector. For our explanation, we designate ambiguous values with the? symbol. One can view an ambiguous inheritance value as a set of values, so 〈0, 0〉? = 〈1, 1〉? = {〈0, 0〉, 〈1, 1〉}. For the earlier example with a previous inheritance value of 〈0, 0〉, the resulting inheritance value would be 〈0, 1〉?. The use of these ambiguous values effectively merges the exponential number of states that would otherwise result. Merging the states in this way suffices because (1) Hapi can later resolve which of the unambiguous inheritance vectors is optimal, and (2) the number of recombinations remains the same regardless of which unambiguous inheritance vector ultimately results. If the previous inheritance value is itself ambiguous, the resulting value must also be ambiguous, and when there is a recombination, the resulting value is unequal to the previous value, such as with 〈0, 0〉? and 〈0, 1〉?.

Hapi resolves ambiguous inheritance values for a state during the back tracing process. While back tracing, if the system encounters a state that has one or more ambiguous inheritance values, it compares these values to the corresponding values at the next (already resolved) locus. If the unambiguous form of this value (that is, that without the ? symbol) or its opposite is equal to the inheritance value at the next locus, the system assigns the equivalent value at the current locus. If neither is equal, recombinations occur on either side of this locus and the inheritance value is truly ambiguous. In this rare case, Hapi's output reports the child's haplotype at this locus as ambiguous.

This optimization significantly improves Hapi's efficiency. Removing this optimization would cause the number of states to grow unwieldy whenever Hapi encountered a locus that has heterozygous parents with the same genotype. Even with all the other optimizations in place, the increase in the number of states would propagate to subsequent loci that have one parent that is heterozygous and the other homozygous.

### State transitions between loci

In general, any state at a previous locus can transition to any state at the next locus. However, because Hapi does not consider state transitions that include recombinations from a parent that is homozygous, and because it uses ambiguous inheritance values, the number of possible state transitions is limited. The state transitions optimization actually comes as a by-product of the two optimizations we have already outlined, yet the effects of these optimizations on the complexity of state transition calculations merit a separate discussion.

At each locus, Hapi considers transitions from the states at the previous locus to either two or four states. If only one parent is heterozygous at the locus, each state at the previous locus can transition to only two states at the current locus. These two states correspond to the two possible phase assignments for the heterozygous parent. A particular phase assignment for the heterozygous parent uniquely defines the inheritance vector bits that that parent transmits. The system copies the other inheritance vector bits from the previous state.

If both parents are heterozygous at a locus, then the parents have four possible phase assignments, and each state at the previous locus can transition to four states at the next locus. The ambiguous inheritance vector optimization makes this possible, since loci in which both parents have the same heterozygous genotype would otherwise produce an exponential number of states. Instead, for a given phase assignment for the parents, a state at previous locus uniquely determines the inheritance vector it transitions to. If the parents are heterozygous with differing genotypes, the children's genotypes at the locus unambiguously imply the complete inheritance vectors corresponding to each parent phase assignment. Thus, exactly four inheritance vectors are possible, and each previous state can transition to these four states.

The efficiency gains of our approach are significant. Without these optimizations, haplotyping algorithms must consider all possible state transitions between loci. If two adjacent loci each have *s *states, other algorithms compute transition probabilities corresponding to all *s*^2 ^state transitions. Use of a fast Fourier transformation reduces the computational burden of these optimizations from a quadratic *O*(*s*^2^) to *O*(*s*·log *s*) [30]. With Hapi's optimizations there are only 2*s *or 4*s *possible transitions, so the computational burden is linear, O(*s*). The speed of computing state transitions - in addition to and in connection with tracking of very few states at each locus - enable Hapi to perform haplotyping calculations very efficiently.

### Equivalent states

At many loci, it is possible to unambiguously deduce which allele each heterozygous parent transmitted to each child. In that case, the inheritance bits that correspond to transmissions from this parent can take on exactly two values depending on the parent's phase assignment. The inheritance bits in these two values are opposite each other, since the parent transmits the same allele in each case, but the alleles reside on opposite homologs for the opposite phase assignments. The locus in Table 3 illustrates these ideas. For this locus, it is easy to deduce which alleles the heterozygous parent transmitted to each child. As well, the two states have opposite inheritance values corresponding to this parent, consistent with their opposite phase assignments.

Two inheritance vectors with opposite bits corresponding to one parent and equivalent bits for the other parent are equivalent in terms of the number and locations of recombinations that will occur at downstream loci. Hapi uses inheritance vectors to detect recombinations. A recombination occurs when the homolog a parent transmitted to a child differs between two loci. Because the parent's inheritance values in these states are exactly opposite each other, each of these inheritance vectors encodes the same set of children as receiving a given homolog. The two states merely use opposite labels for the homologs as implied by the parent's opposite phase assignments. Choosing one of the states instead of the other results in all downstream loci having opposite phase assignments for the parent, consistent with the chosen phase assignment in the upstream locus. The number and location of downstream recombinations are the same regardless of which state the system chooses at this locus because the sets of children that share a common homolog same between states. The two states *a *and *b *in Table 3 are equivalent, and Hapi retains only state *b *and eliminates state *a *from further consideration.

In general, any states with opposite inheritance values for one parent and either equivalent or opposite inheritance values for the other parent are equivalent. This means that, if both parents are heterozygous with differing genotypes, there are only four possible states, and these have equivalent downstream affects. When two or more states are equivalent at a locus, Hapi only retains the state with the fewest recombinations.

Kruglyak *et al*. [22] first discovered a more general form of this optimization, finding that equivalent states exist for all founders in a pedigree. A founder is an individual with no parents in the pedigree. For each founder, the number of inheritance vectors is decreased by a factor of 2. So, whereas there are 2^2*n *^possible inheritance vectors in a pedigree, where *n *is the number of non-founders, this optimization reduces the state space to 2^2*n-f *^inheritance vectors, where *f *is the number of founders. For a nuclear family, *f *= 2, so this optimization reduces the state space by a factor of 4.

### States consistent with Mendel's laws

Although there are 2^2*c *^possible inheritance vectors for every locus, the genotypes of the individuals at a locus often make many of these inheritance vectors inconsistent with the Mendelian laws of inheritance. For example, a parent that has a genotype of *a*/*b *cannot transmit its *b *allele to a child with genotype *a*/*a*. Hapi builds states based explicitly on the genotypes at each locus and spends no time processing any inheritance vectors that are inconsistent with Mendelian inheritance. Merlin [20], GENEHUNTER [21], and Allegro [24] all contain similar optimizations to this, though each spends some small amount of time considering inconsistent inheritance vectors.

### Locus types

Hapi's optimizations apply in different contexts, and in particular, we have identified four types of loci with different parents' genotypes for which different technical issues arise and different optimizations apply. Table 4 summarizes these locus types, listing the number of states that result at each type if there are s states at the previous locus. The table also includes the average number of states that occur at relevant locus types for the dataset we evaluated in Results. (See Additional file 1 for a detailed analysis of Hapi's runtime complexity in general.)

**Table 4:** Four types of loci Hapi distinguishes

			Number of states	
			
Locus type	Parent *p*	Parent *q*	If *s *previous states	Average
Fully informative for both parents	*a*/*b*	*a*/*c *or *c*/*d*	1	N/A
Fully informative for one parent	*a*/*b*	*a*/*a *or *c*/*c*	After informative for parent *q*: 1 Previous states unambiguous: ≤*s* Previous states ambiguous: ≤2*s*	1.87
Partly informative	*a*/*b*	*a*/*b*	≤4*s*	6.31
Uninformative	*a*/*a*	*a*/*a *or *b*/*b*	0	N/A

The four types of loci our algorithm handles separately with the names we use to refer to them. The table lists the number of states that Hapi produces for each type if there are *s *states at the previous locus, and gives the average number of states produced for haplotyping the dataset we evaluate in Results. Note that either parent may have the genotypes listed for parents *p *and *q*.

Loci that we term to be fully informative for both parents are those in which both parents are heterozygous but with differing genotypes. In this case there are exactly four possible states and the equivalent states optimization enables Hapi to retain only one state. Note that although this locus type is advantageous, most SNPs are bi-allelic, and therefore this locus type will not occur in SNP genotype datasets. A fully informative for one parent locus is one that has one heterozygous parent and one homozygous parent. Two successive loci that are fully informative for each of the parents is analogous to one fully informative for both parents locus. Each such locus produces only two possible inheritance vector values corresponding to each parent and, at the second locus, the states are all equivalent.

Often the states at the locus preceding a fully informative for one parent locus do not contain ambiguous inheritance values. When that is the case, because Hapi does not introduce any states with recombinations for the homozygous parent, and the because of the equivalent states optimization, the system retains at most s states. We discuss the case in which the previous locus has states with ambiguous inheritance below. Partly informative loci are those in which both parents are heterozygous with the same genotype. The number of states at these loci may increase by a factor of four from the previous locus, but typically the number of states does not grow large. As Table 4 shows, the average number of states Hapi produces at partly informative loci when haplotyping a real dataset is only 6.31.

Uninformative loci are those in which both parents are homozygous, and yield no information about meiosis. Hapi does not produce any states for these loci, and only deduces the children's phase if they are heterozygous.

### Ambiguous inheritance values and fully informative for one parent loci

Ambiguous inheritance values complicate the handling of fully informative for one parent loci. At this locus type, we apply an optimization to propagate the inheritance vector bits for the homozygous parent from the previous locus. This requires only copying in the case of unambiguous inheritance values, and results in two equivalently labeled states.

The situation is different when a previous state has children with ambiguous inheritance values. In that case, the corresponding two inheritance vectors that Hapi builds are not equivalent because, for children with ambiguous inheritance values, the homozygous parent's inheritance bits are opposite each other rather than equivalent. At the same time, the homozygous parent's inheritance bits for any unambiguous children remain identical across the two values.

Consider the example in Table 5 which is modified from the example in Table 3 to include ambiguous inheritance values. As usual, the inheritance vector values for the heterozygous parent are opposite in the two states. However, the ambiguous inheritance bits correspond to two entirely opposite values, so the two resulting states do not have identical inheritance vector values for the homozygous parent (we underline these differing values in the table). Because the two inheritance vectors are not equivalent, the algorithm cannot eliminate one of these two states. Even so, because the heterozygous parent's inheritance values are still exactly opposite, if the next locus is fully informative for the other parent, Hapi can produce one state at that locus.

**Table 5:** States at a fully informative for one parent locus built from a state with ambiguous values

		***p***_**0**_	***p***_**1**_	***c***_**0**_	***c***_**1**_	***c***_**2**_	***c***_**3**_	***c***_**4**_	# Rec
Prev	$\vec{v}$			〈0, 1〉	〈1, 1〉?	〈1, 1〉	〈0, 0〉?	〈1, 1〉	0
State	*hap*	〈a, g〉	〈a, a〉	〈g, a〉	〈a, a〉	〈a, a〉	〈a, a〉	〈a, a〉	4
*a*	$\vec{v}$			〈**1**, 1〉	〈**0**, 0〉	〈**0**, 1〉	〈0, 0〉	〈**0**, 1〉	
State	*hap*	〈g, a〉	〈a, a〉	〈g, a〉	〈a, a〉	〈a, a〉	〈a, a〉	〈a, a〉	1
*b*	$\vec{v}$			〈0, 1〉	〈1, 1〉	〈1, 1〉	〈**1**, 1〉	〈1, 1〉	

An example, modified from Table 3 and not from real data, showing a state with ambiguous inheritance values (marked by ?) at the previous locus, and the two states Hapi builds based on it. For unambiguous children's inheritance vector values, the system copies the bits corresponding to the homozygous parent from the previous state. For ambiguous children, two opposite inheritance values are valid for the previous state, and the system uses the homozygous parent bit from the inheritance value that matches the heterozygous parent's bit in the state being built. Both of the two inheritance values are necessarily represented, one in each of the resulting states. As the underlined values show, the inheritance values for the homozygous parent differ across the two outputs. As such, the states are not equivalent, and Hapi cannot eliminate either. Bold values indicate recombinations.

Ambiguous inheritance values in states at a locus preceding a fully informative for one parent locus can produce double the number of states at that locus, but does not always do so. While the two inheritance vectors produced by a particular previous state with ambiguous inheritance values are not equivalent, some other previous state may yield an inheritance value that is equivalent to one of these states, thereby enabling the elimination of some states.

### Initial state

To build the initial state from which to haplotype a given chromosome, Hapi uses either a fully informative for both parent locus or two loci that are fully informative for opposite parents. Hapi begins at the first locus on a chromosome, scanning for these types of loci, and skips any partly informative loci. Later, after defining an initial state and haplotyping the remainder of the chromosome, Hapi resolves haplotypes at these early partly informative loci by performing reverse haplotyping from the locus that established the initial state.

A locus that is fully informative for both parents completely defines an initial state. This locus type has exactly four possible inheritance vectors, and because they are equivalent, Hapi arbitrarily chooses one of them.

A fully informative for one parent locus defines half of an inheritance vector, giving information only for the bits that correspond to the heterozygous parent. Hapi again arbitrarily chooses one of the two possible values to assign. The initial state is then partially defined with values for the heterozygous parent. Later, when the system encounters a locus that is fully informative for the undefined parent (or a locus fully informative for both parents), it fills in the inheritance vector values for the undefined parent, and haplotyping proceeds forward normally from this point. The system handles any intervening loci that are fully informative for the already-defined parent in the normal way, while still leaving the homozygous parents' inheritance vector bits undefined. Table 6 (described in more detail below) gives an example of aninitial state defined from two fully informative loci (numbered 8 and 12).

**Table 6:** Example haplotype inference across a series of loci from real data

					**Locus**		***p***_**0**_	***p***_**1**_	***c***_**0**_	***c***_**1**_	***c***_**2**_	***c***_**3**_	***c***_**4**_	**# Rec**					
										
					8	*hap*	〈a, a〉	〈a, c〉	〈a, a〉	〈a, c〉	〈a, c〉	〈a, c〉	〈a, a〉	0					
						$\vec{v}$			〈-, 0〉	〈-, 1〉	〈-, 1〉	〈-, 1〉	〈-, 0〉						
					12	*hap*	〈g, t〉	〈t, t〉	〈t, t〉	〈t, t〉	〈g, t〉	〈t, t〉	〈t, t〉	0					
						$\vec{v}$			〈1, 0〉	〈1, 1〉	〈0, 1〉	〈1, 1〉	〈1, 0〉						
										
	
14	*hap*	〈c, a〉	〈c, a〉	〈a, c〉	〈a, a〉	〈c, c〉	〈a, c〉	〈a, c〉	2	14	*hap*	〈c, a〉	〈a, c〉	〈a, c〉	〈a, a〉	〈c, c〉	〈a, c〉	〈c, a〉	3
	$\vec{v}$			〈1, 0〉	〈1, 1〉	〈0, **0**〉	〈1, **0**〉?	〈1, 0〉			$\vec{v}$			〈1, **1**〉?	〈1, **0**〉	〈0, 1〉	〈1, 1〉	〈**0**, 0〉?	
16	*hap*	〈a, a〉	〈g, a〉	〈a, g〉	〈a, a〉	〈a, g〉	〈a, g〉	〈a, a〉	3	16	*hap*	〈a, a〉	〈a, g〉	〈a, g〉	〈a, a〉	〈a, g〉	〈a, g〉	〈a, a〉	3
	$\vec{v}$			〈1, 0〉	〈1, 1〉	〈0, 0〉	〈1, 0〉	〈1, **1**〉			$\vec{v}$			〈1, 1〉	〈1, 0〉	〈0, 1〉	〈1, 1〉	〈0, 0〉	
17	*hap*	〈t, c〉	〈c, c〉	〈c, c〉	〈c, c〉	〈t, c〉	〈c, c〉	〈t, c〉	4	17	*hap*	〈t, c〉	〈c, c〉	〈c, c〉	〈c, c〉	〈t, c〉	〈c, c〉	〈t, c〉	3
	$\vec{v}$			〈1, 0〉	〈1, 1〉	〈0, 0〉	〈1, 0〉	〈**0**, 1〉			$\vec{v}$			〈1, 1〉	〈1, 0〉	〈0, 1〉	〈1, 1〉	〈0, 0〉	

An example from the real dataset described in Results. The loci are from chromosome 3 and we number them sequentially in the order they occur physically. For simplicity and conciseness, we omit uninformative loci and one non-recombinant fully informative locus between locus 8 and 12. Bold inheritance vector values indicate recombinations. Each state lists its total number of recombinations. Note that the state at locus 14 with minimum recombinations is ultimately not minimum-recombinant globally. See the Example subsection for a detailed description of this table.

### Missing data

Missing genotype data can result either because of quality control mechanisms associated with genotyping technologies or because of non-Mendelian errors (which can be removed using various software packages [33,34]). To handle loci that have children with missing data, Hapi copies the inheritance vector values corresponding to those children from the state(s) at the previous locus to the states at the current locus. This approach assumes a lack of recombination for that child, which is analogous to assuming no recombination at loci where a parent is homozygous. Because the inheritance vector values for that child will no longer be opposite each other between states with opposite parental phase - but will instead be identical - Hapi cannot eliminate states at fully informative loci in the way it does when no data is missing. However, it is still possible to eliminate states in most cases.

The following constitutes Hapi's sixth and final optimization. Consider a set of states that have equivalent inheritance vectors when the missing data children are ignored and with identical inheritance values for those missing data children (that is, states built based on the same previous state). Let *x *be the number of children with missing data, and let *r *be the value of the smallest number of recombinations among this set of states. The states in this set are *x *or 2*x *recombinations away from having equivalent inheritance vectors, depending on whether the inheritance values are opposite each other for transmissions from one or both of the parents. (Viewed another way, if two states have the same assignment of alleles to homologs for one parent and opposite assignments for the other, the inheritance vectors are *x *recombinations away from being equivalent. If both parents have opposite allele assignments, the inheritance vectors must be entirely opposite each other and therefore 2*x *recombinations separate them since the missing data children's inheritance values are identical, not opposite.) Considering states that are separated by *x *recombinations, a state that has more than *r *+ *x *recombinations will always be less optimal than the minimal state and can therefore be removed. Even if all the missing data children later recombine relative to the state with *r *recombinations - which would produce an inheritance vector equivalent to the larger state - that minimal state would yield *r *+ *x *recombinations - that is, fewer than that for the larger state.

Although this technique will not always eliminate the same number of states as if full data were available, it is quite effective. Our experimental results demonstrate this as Hapi very efficiently analyzes a real dataset that includes missing data (see Results). Often one state at a locus will have zero or one recombinations compared to another state that has all or all but one child recombining. In such a case, the technique just described will typically be able to eliminate the state with more recombinations.

Hapi does not currently handle loci that are missing data for one or both parents. It can be modified to do so by building states corresponding to all possible parent genotypes consistent with the children's genotypes [36].

### Example

We give a brief example illustrating some aspects of our algorithm in Table 6. This example is from real data for one of the families in the Huntington's Disease Venezuela Collaborative Study [32] dataset discussed in Results. The initial locus 8 defines inheritance vector values for parent 1, the heterozygous parent, but leaves the values for parent 0 undefined (designated by -). When analyzing this example, Hapi produces a complete initial state at locus 12, where it deduces inheritance vector values for parent 0 and copies those for parent 1 from locus 8. (Note: this table omits uninformative loci.) Locus 14 is partly informative, and with one state at the previous locus, it has only four states corresponding to the four possible parents' phase assignments. The table shows two of these four states, one on the left and one on the right. The two omitted states have four and five recombinations at locus 14 and still more at locus 16 and 17.

The left side state at locus 14 has two recombinations. It transitions to two states at locus 16, one with a total of three recombinations and one with five; the table shows the state with fewer recombinations. These two states at locus 16 both transition to the same two states at locus 17, and we include the state with fewer recombinations in the table.

The right side state for locus 14 has three recombinations. Although this is greater than the two local recombinations shown for the left side state, this state actually yields fewer recombinations globally. It transitions to two states at locus 16, one of which produces no additional recombinations, and likewise that non-recombinant state produces zero recombinations at locus 17. This path of states therefore has only three recombinations, which is minimal across these loci.

Although this discussion considered the downstream effects of each state at locus 14 separately, Hapi considers all states at successive loci at the same time and does not revisit loci. The four states at locus 14 each transition to two non-equivalent (because of ambiguous inheritance values) states at locus 16, for a total of eight states. Because locus 16 is fully informative for parent 1, the inheritance vector values for that parent are equivalently labeled in these states. Locus 17 is heterozygous for parent 0 and produces exactly four equivalently labeled states, and the state with the fewest recombinations must be globally minimal. This globally minimal state is on the right side of the table.

### Maximum likelihood haplotyping

We now formulate the problem of maximum likelihood haplotyping and show how to solve it using the same techniques as those we employ for minimum-recombinant haplotyping.

Suppose we have genotyped loci numbered 0,...,*L *for each member of a nuclear family with *c *children, and assigned inheritance vectors $\vec{v}$ for each locus *l*. Let *θ_l _*be the recombination frequency between locus *l *and *l *- 1 for all 0 <*l *≤ *L*. Also let $r\left(l\right)=H\left({\vec{v}}_{l-1},{\vec{v}}_{l}\right)$, the number of recombinations (Hamming distance) between the inheritance vectors at loci *l *- 1 and *l*. Then the probability of the assigned inheritance vectors is:

(2) $$P=\prod_{l=1}^{L}\theta_{l}^{r(l)}\cdot {\left(1-\theta_{l}\right)}^{2c-r(l)}.$$

Using log likelihoods, this can be written as:

(3) $$ℒ=\sum_{l=1}^{L}\ln(\theta_{l})\cdot r(l)+\ln(1-\theta_{l})\cdot [2c-r(l)].$$

This formulation of the maximum likelihood problem shows clearly the relationship of the maximum likelihood problem to the minimum-recombinant one. If all loci have the same recombination frequency *θ *< 0:5, then the maximum likelihood solution is the same as the minimum-recombinant one since ln(*θ*) < ln(1 - *θ*) across all loci, so decreased *r*(*l*) values increase the overall likelihood. However, when the recombination frequencies differ across loci, more recombinations at one locus may have higher likelihood than fewer recombinations at another.

A dynamic programming equation computing maximum likelihood haplotypes can be written as follows, where *l *is a locus, and $\vec{v}$, $\vec{w}$ are inheritance vectors:

(4) $$P(l,\vec{v})=\underset{\vec{w}}{\max}\{P(l-1,\vec{w})\cdot \theta_{l}^{H(\vec{w},\vec{v})}\cdot {\left(1-\theta_{l}\right)}^{2c-H(\vec{w},\vec{v})}\}.$$

Using log likelihoods, the dynamic programming formulation becomes:

(5) $$L(l,\vec{v})=\underset{\vec{w}}{\max}\{L(l-1,\vec{w})+\ln(\theta_{l})\cdot H(\vec{w},\vec{v})+\ln(1-\theta_{l})\cdot [2c-H(\vec{w},\vec{v})]\}.$$

Immediate application of the above formula is problematic because we cannot completely ignore uninformative loci: they have non-zero recombination frequencies that affect the overall probability of a solution. Without some novel insight, it is necessary to model most or all of the 2^2c^/4 non-equivalent inheritance vectors at uninformative loci, and at least 2^c^/2 inheritance vectors at loci that are fully informative for one parent.

In order to account for recombination frequencies at loci where both parents are homozygous - that is, at uninformative loci - Hapi computes modified recombination frequencies at all other informative loci, including fully informative for one parent loci where one parent is homozygous. These modified recombination frequencies include the recombination frequencies for all uninformative loci that occur between a given informative locus and the nearest upstream informative locus. In calculating these probabilities, the algorithm is pre-computing the effects of recombination frequencies at uninformative loci, allowing it to avoid directly processing such loci.

We denote Hapi's modified recombination frequency at a locus *l *as *φ_l _*and the frequency of non-recombination (expressed above as (1 - *θ_l_*)) as *ψ_l_*. To calculate *φ_l _*and *ψ_l _*for a locus *l*, let *l*_0 _be the nearest upstream informative locus and let *l*_1, ..., _*l*_*n*-1 _be the uninformative loci that appear between *l*_0 _and *l*. Let *l*_n _= *l *and let l* be the locus with the highest recombination frequency, that is, find l* ∈ {*l*_1_, ..., *l_n_*} such that $\theta_{l^{*}}=\max_{i=1}^{n}\theta_{l_{i}}$. Then:

(6) $$\phi_{l}=\theta_{l*}\cdot \prod_{i=1,l_{i}\neq l*}^{n}\left(1-\theta_{l_{i}}\right).$$

Thus, the probability of recombination between locus *l*_0 _and *l*_n _= *l *is equal to the maximum between-marker recombination frequency within the region spanned by these loci, or θ_l*_, multiplied by the probability of not recombining anywhere else. Note that *θ*_l* _is the probability of recombining between locus l* and l* - 1, and either or both of these loci can be uninformative. Hapi stores the locus number l* so that the final haplotype solution includes any recombinations in their most likely positions.

A consequence of this formula is that at most one recombination can occur between any two informative loci on a given homolog. Thus, within a region of uninformative loci, we do not model the possibility of intervening gene conversions or double recombinations. Not modeling such events is sensible because it is impossible to observe or verify them. Furthermore, haplotypes that include additional recombinations or gene conversions not directly implied by the data are less likely than those without these events since *θ_l _*< 0.5 means recombination is less likely than non-recombination. Therefore, even if we were to model such events, they would not ultimately appear in the haplotype solution, so we lose nothing by not modeling them.

The probability of not recombining between locus *l*_0 _and *l_n _*= *l *is the product of non-recombination across each of the locus intervals:

(7) $$\psi_{l}=\prod_{i=1}^{n}\left(1-\theta_{l_{i}}\right).$$

The equations for *φ_l _*and *ψ_l _*utilize the recombination frequencies between each pair of loci rather than a single recombination frequency spanning the region between *l*_0 _and *l*. This is the case because the haplotyping output must place every recombination at some discrete location between a pair of markers. There must exist a pair of markers flanking every recombination, and sometimes one or both of these will be uninformative. This matches the maximum likelihood approach employed by other algorithms, which calculate the probability of recombining (or not) between each pair of markers, not just those that are informative. A consequence of this formulation is that *φ_l _*+ *ψ_l _*≠ 1. This occurs because, as we earlier noted, these probabilities account for the possibility of only one recombination on a given homolog between any two informative loci - more than one recombination will always be less likely. Some applications - notably linkage analysis; see below - may benefit from using a single recombination frequency between the region spanned by *l*_0 _to *l*. Our algorithm functions the same regardless of how we calculate *φ *and *ψ*. To increase numerical stability and efficiency, Hapi uses the log likelihood formulation of this dynamic programming problem. This formula substitutes multiplication for exponentiation and uses the values ln(*φ_l_*) and ln(*ψ_l_*) which requires summation instead of multiplication to calculate. The dynamic programming equation for maximum log likelihood haplotypes at a locus *l *is thus given by the following:

(8) $$L(l,\vec{v})=\underset{\vec{w}}{\max}\{L(l-1,\vec{w})+\ln(\phi_{l})\cdot H(\vec{w},\vec{v})+\ln(\psi_{l})\cdot [2c-H(\vec{w},\vec{v})]\}.$$

The formulation just presented solves the problem of needing to track states at uninformative loci, but does not resolve another important issue. One of Hapi's key optimizations is to avoid modeling states that exhibit recombination from a parent that is homozygous at a locus, including at fully informative for one parent loci. This approach suffices in order to produce minimum-recombinant haplotypes since recombinations only occur at the informative locus that reveals them. For maximum likelihood haplotyping, if some informative locus exhibits recombination, the most likely location of that recombination might be upstream of an earlier fully informative for one parent locus where the transmitting parent is homozygous.

A further complication to this issue of fully informative for one parent loci is the interactions between such loci and partly informative loci. A child may exhibit an ambiguous recombination at a partly informative locus immediately after a fully informative for one parent locus. In this case, the recombination might occur at the partly informative locus or upstream of the fully informative for one parent locus on the homozygous parent's homolog. Confounding this issue is the possibility of additional recombinations occurring downstream of the partly informative locus in this scenario. Although we could immediately evaluate the relative likelihoods of placing the ambiguous recombination upstream or at the partly informative locus, a downstream recombination may affect the overall likelihood.

Complicated dependence across loci can also occur when a fully informative for one parent locus appears downstream of a partly informative locus where the child is heterozygous. The earlier partly informative locus may be the result of an ambiguous recombination and the opposite inheritance value may have equivalent or nearly equivalent likelihood. Choosing the opposite inheritance at the partly informative locus introduces a recombination but also inverts the inheritance value that occurs for the homozygous parent at the subsequent fully informative for one parent locus. Making such a change affects the inheritance values at downstream loci and may be more likely than some other downstream recombination. To address all these possibilities, Hapi tracks the probability of an *alternate inheritance *value for each child at a locus. In the case of fully informative for one parent loci, this is the probability of inverting the inheritance value transmitted by the homozygous parent. For partly informative loci, the alternate inheritance has inverted homolog transmissions from both parents. The alternate inheritance probability at a locus depends on the surrounding loci. For example, the system can assign the alternate inheritance at a fully informative for one parent locus by recombining at that locus or, if the previous locus is fully informative for the same parent or partly informative, the system can apply the alternate inheritance (and associated probability) at the previous locus. (Note that the system must account for any additional local recombinations introduced by using the alternate inheritance at the previous locus.) Hapi evaluates the probabilities for all possible ways of assigning the alternate inheritance for each child at a locus and stores those probabilities and the information relating to how they are assigned. Because of space considerations, we omit the details of how Hapi handles alternate inheritance probabilities; a complete description is available in another document [36].

We note briefly that because the alternate inheritance probabilities will differ across states at a locus, whenever multiple states transition to the same state at the next locus, Hapi must sometimes track multiple probabilities. To do so, it stores a range of probabilities - the maximum and minimum alternate probability for each child across all previous states. The alternate probability is therefore ambiguous during haplotyping and can only be determined during back tracing as Hapi explores the paths yielding the alternate inheritance values. Determining the maximum likelihood haplotyping assignment therefore requires back tracing to determine which path of states yields the highest likelihood and then forward tracing to assign those states.

### Linkage analysis and LOD scores

The basic Hapi algorithm is applicable not only to haplotype reconstruction but also to linkage analysis and LOD score calculations. This paper provides an overview of how to apply Hapi to linkage analysis; for more details, see Williams [36]. Briefly, all that is required is that Hapi retain all states it considers across all loci (that is, it should not perform back tracing), and that it calculate state probabilities that fully account for inheritance values at both upstream and downstream loci. Hapi already includes probabilities for upstream loci in each state. To include the probabilities of states at downstream loci, Hapi must perform a second traversal of all the loci/states in reverse, multiplying a given state's probability by the appropriate transition probability and the probability of the state at the next locus. With the probability distribution of locus states computed in this way, Hapi can calculate LOD scores (or non-parametric Z scores) by weighting the score of each possible inheritance vector by its probability at the locus in question [22]. Hapi does not consider all possible states at each locus; specifically, it omits any states that exhibit double recombinations within a series of uninformative loci. Hapi also omits some states at partly informative loci, depending on the inheritance values at surrounding loci. Any omitted states always have lower likelihood than some other included state, and will typically exhibit extra recombinations and thus have considerably lower likelihood. Because of their lower likelihood, the potential impact on the overall linkage score of any omitted states is proportionately limited. As well, a high LOD score for a state that exhibits a double recombination is suspect since physical limitations make double recombinations within a short span of uninformative loci extremely unlikely. To more fully account for possible inheritance vector assignments at partly informative loci, Hapi could detect and include states in which the opposite inheritance assignment for a child or children does not yield additional recombinations but only places the recombinations differently across parents. Making this change for partly informative loci would enable Hapi to omit only states that exhibit additional recombinations relative to those it already considers. This change would serve to exclude very unlikely states from consideration and include marginally likely ones.

Hapi's dramatic efficiency gains over other algorithms make it attractive to apply to linkage analysis for disease gene studies. Its optimizations make possible analyses of datasets for which current algorithms either fail or have significant time and storage requirements.

## Abbreviations

HMM: Hidden Markov Model; LOD score: Logarithm base 10 of Odds score; MTBDD: Multi-Terminal Binary Decision Diagram; SNP: Single Nucleotide Polymorphism.

## Authors' contributions

ALW devised and implemented the Hapi algorithm and wrote this paper under the supervision of DEH, MCR, and DKG.

## Supplementary Material

Additional file 1 **Analysis of Hapi's runtime complexity**. A detailed discussion of Hapi's runtime complexity, including descriptions of inputs that can yield runtime that is exponential in the size of the family. Also a probabilistic analysis of the likelihood of one class of these inputs occurring in real data. Click here for file
