# Supplementary material for: Rapid haplotype inference for nuclear families
Source: Genome Biol. 2010 Oct 29;11(10):R108. doi: 10.1186/gb-2010-11-10-r108 (PMC3218664; doi:10.1186/gb-2010-11-10-r108)
Supplement: Additional file 1 — Analysis of Hapi's runtime complexity. A detailed discussion of Hapi's runtime complexity, including descriptions of inputs that can yield runtime that is exponential in the size of the family. Also a probabilistic analysis of the likelihood of one class of these inputs occurring in real data. [file gb-2010-11-10-r108-S1.pdf]

Additional file 1

As we discuss in Results, Hapi is extremely efficient, running orders of magnitude faster than existing algorithms. These experimental results demonstrate that Hapi is very efficient in practice. If L is the number of marker loci, s is the maximum number of states produced at any locus, and c is the number of children, the runtime complexity of Hapi's minimum-recombinant algorithm is $O(L \cdot s \cdot c)$. This is so because the algorithm visits each of the L loci at most two times (with a possible second visit during back tracing), and at each locus, Hapi builds $O(s)$ states, and each state has size $O(c)$. While Hapi is efficient in practice, there are two rare corner cases where $s = O(2^{2c})$. That is, the number of states can be exponential in the number of children in the family being analyzed. We explore these possibilities in more detail below.

Hapi's maximum likelihood algorithm is more complicated than the minimum-recombinant algorithm because it must resolve cases in which the optimal location or presence of an alternate inheritance assignment is ambiguous. The maximum likelihood algorithm does this during back tracing, and in the worst case, it must explore an exponential number of state paths that correspond to many possible ways of assigning alternate inheritance across the loci. In practice, because alternate inheritance only applies to homologs transmitted by uninformative parents or to heterozygous children at partly informative loci, the extent to which alternate inheritance can increase the complexity of back tracing is limited. The algorithm need only track a possible alternate inheritance assignment for a child until it encounters a fully informative for both parents locus (including two loci fully informative for opposite parents or a partly informative locus where the child is homozygous). Our experimental results demonstrate that the maximum likelihood version of Hapi is extremely efficient despite the potential for an exponential runtime.

As noted above, there are corner cases in which the number of states in Hapi can grow to be exponential. Because of the nature of the requirements for these corner cases to occur, they are extremely unlikely to happen in practice. As well, if a series of loci do exhibit one of these cases, the number of states becomes exponential only transiently because a later locus that is fully informative for both parents and with data for all the children (or two successive loci fully informative for opposite parents) will produce a single state. One necessary condition for this state blowup is therefore that the series of loci not contain such a locus (or loci) that produces a single state.

The first exponential case arises because of missing data. Consider a fully informative for one parent locus where the previous locus has s states. If one or more children have missing data at this locus, the number of states for this locus, which would otherwise be s (absent previously ambiguous inheritance values), can instead become $2s$. In order for this increase in states to occur, a large proportion of the children must

have missing data, or a large proportion must recombine. Without these conditions, the optimization described in the main text would apply. These conditions are unlikely in practice since recombination is rare, but one can construct such a pathological input. Note that if a child that was missing data at some previous locus has data at the current locus, states that were added at the previous locus effectively merge at the current locus.

The above properties indicate the possibility, however remote, for Hapi to produce an exponential number of states from loci with missing data. Consider a series of loci where each successive locus has missing data for the same set of children at the previous locus as well as missing data for one more child. (That is, the first locus has data for all children, the second missing data for child c_0, the third missing data for child c_0 and c_1, etc.) This scenario could lead to an exponential number of states, but only if the previously mentioned optimization does not apply. A more complicated scenario in which at least half of the children have missing data at the starting locus would defeat the optimization since the value of x mentioned in the text would be large. However, such a scenario is extremely unlikely to occur in practice. Indeed, our simulation results discussed in the main text indicate that Hapi is able to cope with large proportions (up to 50%) of missing data, as it outperforms Merlin even in this computationally taxing scenario (see Table 2).

The second way an exponential number of states can occur comes through ambiguous inheritance values, which Hapi introduces at partly informative loci. Ignoring additional states that may arise because of missing data, the first encountered partly informative locus will have 4 states. Without any recombination, the maximum number of states that may arise across any number of partly informative loci is 12. This maximum of 12 states occurs because lack of recombination constrains children's inheritance values to be fixed relative to each other. With these constraints, for a particular assignment of parent's alleles to homologs, there are three possible inheritance values for heterozygous children (two non-ambiguous and one ambiguous). Since there are four phase assignments of the parents at these loci, there can be at most $4 \cdot 3 = 12$ states.

When recombination occurs in a child or children between two partly informative loci, the number of states at the second locus increases *additively*, with two additional states for each assignment of parent's alleles (for a total of eight added states). The recombination changes the constraints on possible children's inheritance values and effectively breaks the children into two classes. The children in one class exhibit recombination relative to the other class and have ambiguous inheritance. Until the remainder of the algorithm completes, which of these two classes has fewer recombinations is unknown, so the system must

track additional states (i.e., both classes). In general, since all c children can recombine, this can lead to at most $4(2c+1)$ states, a polynomial number, so the number of states cannot become exponential across a series of loci that are all partly informative.

The number of states can grow to be exponential through interactions between partly informative and fully informative for one parent loci. Whenever a locus has states with ambiguous inheritance values and occurs immediately before a fully informative for one parent locus, the number of states may double, as the text describes. The scenario in which an exponential number of states can occur is when there are a series of alternating partly informative and fully informative for one parent loci. At each partly informative locus, one of the children must recombine and become heterozygous, yielding an ambiguous inheritance value, and that child must remain heterozygous at all successive partly informative loci. The recombination results in an additional $4 \cdot 2$ states at each partly informative locus, followed by a doubling in the number of states at the fully informative for one parent locus. With each child recombining once at some partly informative locus, the number of states doubles c times for an exponential blowup.

The above scenario is extremely unlikely. It requires recombinations to occur in all the children, all at partly informative loci, and without encountering a locus or loci that produce a single output state. Moreover, after recombining, the children must be heterozygous across all partly informative loci. This is unlikely because homozygous and heterozygous genotypes are equally probable for children at such loci. Although this scenario could occur for a small number of children (say two or three), the likelihood decreases as the number of children increases. Thus, when an exponential blowup would be most problematic (for large c), it becomes less likely.

To estimate the probability of this scenario occurring, let $\theta < 0.5$ be the recombination probability between each locus within some series of loci. Let L be the number of loci in this series, and assume that each partly informative locus is followed by a fully informative for one parent locus and vice versa. Then the probability of all c children separately recombining at some partly informative loci within this region is $\binom{L/2}{c} \cdot \theta^c \cdot (1-\theta)^{L \cdot c - c}$, since the recombinations occur at some c out of the $L/2$ partly informative loci, and since there are $L \cdot c$ transitions across all loci with c of these being recombinations. The likelihood of a child being heterozygous across a series of p partly informative loci is $\left(\frac{1}{2}\right)^p$. A set of children that are either all heterozygous or all homozygous at a partly informative locus all receive either the same or exactly opposite homologs from the parents. As a result, when no recombination occurs, these children will continue to all be either heterozygous or homozygous at downstream partly informative loci. It suffices therefore to require only one recombined child to have the necessary heterozygous genotype; the others will

be constrained to be the same because of lack of additional recombination. The following is an upper bound on the probability for this scenario, since it does not account for the likelihoods of the locus types appearing in the required order:

$$P < \sum_{r=c}^{L/2} \binom{L/2}{r} \theta^r \cdot (1-\theta)^{L \cdot c - r} \cdot \left(\frac{1}{2}\right)^r . \quad (1)$$

The summation requires the children to recombine at at least c loci, but optionally at all $L/2$ partly informative loci. The $\left(\frac{1}{2}\right)^r$ term requires that the recombined children be heterozygous at all recombining loci, but a more accurate (and in general lower) probability would require heterozygousity at all loci after the first location that recombines.

Equation 1 has an extremely low probability, even if θ is near 0.5. It is also a conservative estimate, because the children must have the proper inheritance vector values at the locus preceding the locus at which recombination occurs to ensure that the child becomes heterozygous and not homozygous. Additionally, as noted above, it does not account for the requirement that the L loci not contain a fully informative for both parents locus (or two fully informative for one parent loci) that will produce only one state.

This analysis shows the low likelihood of an exponential blowup in the state space in Hapi, demonstrating that real data is extremely unlikely to ever produce large runtimes.
